# JSOM: Jointly-evolving self-organizing maps for alignment of biological datasets and identification of related clusters

**DOI:** 10.1371/journal.pcbi.1008804

**Published:** 2021-03-16

**Authors:** Hong Seo Lim, Peng Qiu

**Affiliations:** Department of Biomedical Engineering, Georgia Institute of Technology and Emory University, Atlanta, Georgia, United States of America; University of South Carolina, UNITED STATES

## Abstract

With the rapid advances of various single-cell technologies, an increasing number of single-cell datasets are being generated, and the computational tools for aligning the datasets which make subsequent integration or meta-analysis possible have become critical. Typically, single-cell datasets from different technologies cannot be directly combined or concatenated, due to the innate difference in the data, such as the number of measured parameters and the distributions. Even datasets generated by the same technology are often affected by the batch effect. A computational approach for aligning different datasets and hence identifying related clusters will be useful for data integration and interpretation in large scale single-cell experiments. Our proposed algorithm called JSOM, a variation of the Self-organizing map, aligns two related datasets that contain similar clusters, by constructing two maps—low-dimensional discretized representation of datasets–that jointly evolve according to both datasets. Here we applied the JSOM algorithm to flow cytometry, mass cytometry, and single-cell RNA sequencing datasets. The resulting JSOM maps not only align the related clusters in the two datasets but also preserve the topology of the datasets so that the maps could be used for further analysis, such as clustering.

## Author summary

Biological datasets are now generated more than ever as many data acquisition technologies have been developed over the years, especially single-cell technologies. With increasing amounts of datasets available for larger scale studies, robust computational tools that could align datasets are needed for data integration and interpretation. We present a new algorithm that can align two biological datasets and demonstrated that the algorithm can work with data generated from different data acquisition technologies. Our proposed algorithm produces low dimensional representations of two datasets to align them in a way that preserves the topology of the respective datasets. Such aligned maps facilitate further analysis, such as clustering. The proposed algorithm showed promising results when applied to different combinations of datasets, i.e., flow cytometry to flow cytometry, flow cytometry to mass cytometry, and two different single-cell RNA sequencing technologies. Therefore, our newly developed algorithm could potentially lead to new discoveries that were once difficult to obtain.

This is a *PLOS Computational Biology* Methods paper.

## Introduction

Rapid advances in technology have led to the generation of a myriad of digital data in various types and formats. Specifically, the biology and medicine field experience the noticeable progress in measurement technology, and biological data can now be generated through many different techniques in a higher resolution than ever [1]. For instance, multiple parameters of individual cells could now be measured not only through the conventional flow cytometry but also through the mass cytometry [2], and the number of parameters measured for both technologies has steadily increased as well. Besides, with the development of next-generation genomic sequencing technologies in recent years, single-cell RNA sequencing (scRNA-seq) is capable of measuring transcriptome at single-cell resolution through various techniques such as Drop-seq, Smart-seq, MARS-seq, CEL-seq, and Seq-Well [3–8].

With the growing amount of available data, extensive studies now require comparisons of multiple datasets that are acquired by potentially different technologies. One of the practical utilities of comparing different datasets is the identification of related data points across different datasets. Related data points could reflect they are of the same cell types, or they are related to the same biological function, such as an expression of a gene and its methylation site, for example. Nevertheless, a challenge arises when comparing datasets generated by different technologies, because the data and features may not be readily compatible. Even for the datasets acquired by the same technology, biological datasets are often inherently affected by variations due to non-biological factors, which is called a batch effect. To address these challenges, there have been many studies to facilitate comparison and integration of different datasets. For instance, various methods were developed to facilitate data comparisons and/or to correct batch effect for flow and mass cytometry data [9–11], and also for microarray and single-cell RNA-seq data [12–16]. Even though they utilized different approaches from one another, many methods share a common objective of enabling a more robust data integration and comparison of the datasets.

Here we present a new method called JSOM, which can align two datasets and identify related clusters from the two datasets. JSOM’s strength is highlighted when it is used to match single-cell datasets generated across different platforms, e.g. between flow cytometry and mass cytometry, or between two different single-cell RNA-Seq technologies. JSOM takes two datasets as inputs. The outputs of JSOM are two aligned maps that capture a low-dimensional representation of the organization of cell clusters shared by the input datasets, and can be used to identify related clusters of the two datasets. The utility of JSOM encompasses both clustering the datasets and finding similar clusters across the two datasets. In addition, when cells in one of the input datasets is annotated with cell type labels, JSOM can be used to perform label transfer, predicting cell type labels for cells in the other input dataset. The proposed JSOM is based on the self-organizing map(SOM) [17], which is an unsupervised technique producing a low-dimensional discretized representation, called a map, of the training samples. SOM is often used for clustering and dimensionality reduction, and its successful usages in biological data have been shown in various studies [18–20]. In the original SOM algorithm, the low-dimensional map is iteratively evolved (updated) to resemble the input space of a single dataset. Our proposed algorithm allows two maps to evolve with respect to two datasets simultaneously so that the resulting maps could be used to interpret how the two different datasets are related through the alignment of the maps. In addition to providing the aligned maps, JSOM also effectively cluster the datasets while preserving the topology of the datasets, the features that the original SOM is known for. Here we will describe in detail how JSOM operates and how the jointly-evolved maps could be used to find related clusters of data across different combinations of datasets.

## Results

### Outline of JSOM using mouse bone marrow flow cytometry data

**Fig 1:**
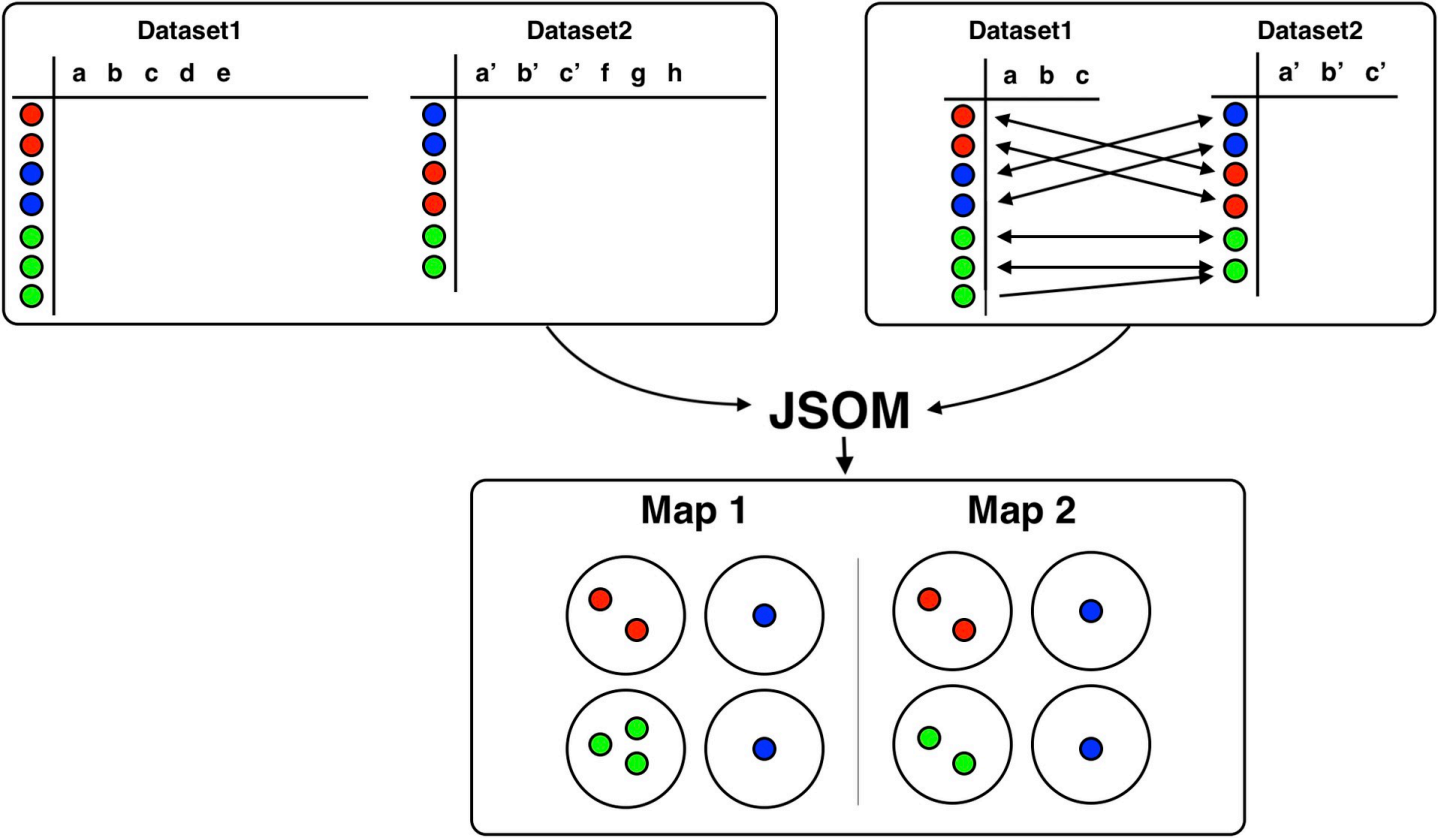
Overview of JSOM. Overview of JSOM. Dataset1 and Dataset2 are two datasets that measured similar type of cells where different color represents different cell types. Among the features measured for the datasets, feature a, b, c from dataset1 are known to correlate with feature a’, b’, c’ from dataset2. Using the related features, matching functions are figured based on Pearson’s correlation using the related features only. JSOM generates two maps where data points in the two datasets are assigned to nodes in the two maps respectively, and two nodes at the same location of the two maps will represent the same cell types in the two datasets.

To demonstrate how JSOM works, we applied it to a mouse bone-marrow flow cytometry dataset from Qiu et al. [21]. Previous manual gating analysis of this dataset used protein markers such as CD11b, c-kit, B220, TCR-B, CD4, and CD8, to identify five distinct cell types: Myeloids, CD4 T Cells, CD8 T Cells, B Cells, and HSPC. We randomly sampled 20,000 cells from the mouse bone marrow dataset and divided the 20,000 cells into two datasets containing 10,000 cells each. We call the two datasets as dataset1 and dataset2. In addition to the datasets, JSOM also requires information about which features in each dataset are shared (or related) between the two datasets. Given the information of the shared features, JSOM evaluate a datapoint-to-datapoint matching function that maps each data point from one dataset to the data point from the other dataset, that is most related. The matching is by default determined based on the Pearson’s correlation coefficient. Now with the two datasets and the mapping function, all necessary inputs for JSOM are ready. A schematic of the JSOM procedure is described in Fig 1.

The operation of JSOM can be divided into three parts. First, JSOM initializes low-dimensional maps using a rectangular grid of nodes. A user could define the number of nodes, and here we use 100 nodes (10 by 10 in a grid) which is the default setting for JSOM. The location of the nodes is equally spread out in the two-dimensional space, which we call a map. Two maps are initialized one for the dataset1 and the other for the dataset2. For each map, nodes are set to have a vector length corresponding to the number of features of respective datasets. For the mouse bone marrow datasets, both datasets have 14 features, and we will call the collection of vectors of length 14 of the first map as the first set of vectors of nodes, and the collections of vectors of the second map as the second set of vectors of nodes.

Second, JSOM iteratively updates the set of vectors of the maps. For each iteration, one data point from dataset1 and one data point from dataset2 are randomly chosen. For the data point drawn from dataset1, its Euclidean distance to the first set of vectors of nodes are calculated. A node with smallest distance is determined. The node that contains the closest vector and all the nodes in the neighborhood are updated so that their corresponding first set of vectors resemble the chosen data point from dataset1. Similarly, the second set of vectors of nodes are updated based on the data point drawn from dataset2. We call the above steps independent updating, because they are identical to the original SOM algorithm.

Joint updating occurs after the independent updating. For the data point drawn from dataset1, its most related data point in dataset2 is determined according to the matching function (e.g., correlation based on the shared features). Then, the node closest to the data point drawn from dataset1 is determined based on Euclidean distances to the first set of vectors, and both the first and second vector sets of nodes of the same location and nearby nodes are updated using the data point drawn from dataset1 and its matched data point from dataset2. The amount of update is proportional to how close, in terms of their correlation, the data point drawn from dataset1 and matched data point from dataset2 are. The same procedure is applied to the data point drawn from dataset2. The above independent and joint updating steps iterate until convergence.

Lastly, once the maps are fully trained, for every data point in dataset1 and dataset2, JSOM find its nearest node among the updated nodes of the two maps and assign the data point to the closest node (i.e., data points in dataset1 are assigned to nodes the first map, and data points in dataset2 are assigned to the second map). The collection of assignments of data points to nodes is the final result of JSOM, and we claim that data points assigned to the same node are related clusters between the two datasets as illustrated in Fig 1. As an optional step, JSOM could superpose two maps and concatenate two vectors of the same location node and perform the hierarchical clustering of nodes using the concatenated vectors.

**Fig 2:**
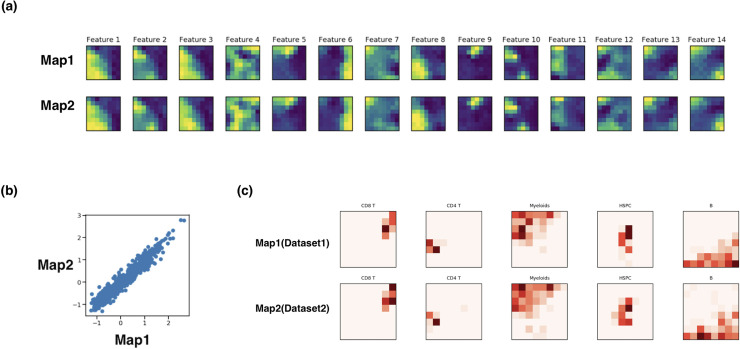
JSOM Results on Mouse Bone Marrow Dataset. (A) Visualizations of each feature of two maps after JSOM training. Alignment is robust for all features. (B) Scatter plot of pairs of same location grid values is shown on the right. Strong linear relationship suggests the maps are well aligned across all features. (C) Visualizations of distributions of various cell types in the two datasets on the JSOM maps. After JSOM training, data points in the two datasets are assigned to the nodes. For cell type, the percentages of its cells assigned to each node are used to generate the heatmaps. Here, the distributions of B, CD4 T, CD8 T, HSPC and Myeloid cells are shown. Notice that each cell type occupies similar region in the grids.

Fig 2A visualizes each feature on the two resulting maps after JSOM was applied to the bone marrow datasets. Visually, the maps were aligned as intended. We further quantified the alignment of the maps through root mean square error of each feature for each grid, which turned out to be 0.0346, suggesting the two maps are quantifiably well aligned as shown in the scatter plot in Fig 2B. We were able to confirm that such well aligned maps led to correct matching of cell types in the two datasets, as shown in Fig 2C, which visualizes where data points of each cell type from the two datasets were assigned to. Cells of the same type occupied similar nodes in both maps, suggesting JSOM’s strong performance in aligning datasets and identification of related clusters. Detailed descriptions of the JSOM algorithm are provided in Methods.

To quantify the effectiveness of JSOM, we devised two metrics called the node purity score and the matching score (see Methods). The node purity score is defined as the average proportion of cells from the same dataset assigned to the same nodes of the JSOM maps that shared the same cell type label, hence quantifying the clustering power but independent of the alignment of the two maps. The matching score is defined as the average proportion of cells from the two datasets belonging to the same nodes that shared the same cell type label, which quantifies the alignment of the two maps. The matching score for the above analysis was 0.926, and the clustering score was 0.950. These scores suggest that JSOM successfully clustered each dataset into distinct cell types, and correctly matched the cell types in the two datasets.

### Flow cytometry and mass cytometry datasets of human PBMC

To provide a challenging yet relevant scenario, we applied JSOM to human peripheral blood mononuclear cell (PBMC) datasets acquired by two different technologies, i.e. flow cytometry and mass cytometry. Both technologies aim to measure physical and chemical characteristics of cells and have research applications in medical field including immunology and hematology. Even with the shared objective of measuring similar characteristics of cells, these two technologies generate noticeably different data manifested by different number of features and different distribution of values measured. Hence, comparing datasets acquired by these two technologies could not be easily done even when the datasets represent measurements of the same biology. JSOM could prove useful in the case like this, where datasets are known to be related (i.e. similar biology) and also where there are ways to identify similar/related features between the datasets. From FlowRepository, we downloaded a flow cytometry dataset (https://flowrepository.org/id/FR-FCM-Z2KP) and a mass cytometry dataset (https://flowrepository.org/id/FR-FCM-ZZWC), and internally identified cell types through manual gating. The flow cytometry dataset contained measurements of 35 protein markers, and five different cell types were identified. Due to the panel of markers not specialized to identify monocytes, dendritic cells, and natural killer cells, the five cell-type groups that could be identified were (1) B cells, (2) CD4 T cells, (3) CD8 T cells, (4) monocytes and dendritic cells grouped together, and (5) the rest of cells that are conjectured to be natural killer cells. The mass cytometry dataset contained measurements of 48 protein markers, and six different cell types were identified. The identified cell types were (1) B cells, (2) CD4 T cells, (3) CD8 T cells, (4) monocytes, (5) dendritic cells, and (6) NK cells. Between the two datasets there were 7 overlapping protein markers. We used these 7 overlapping markers to define the matching function that connects data points in the two datasets. In this specific example, we demonstrate another option for defining a matching function, called ‘binary’. The ‘binary’ option is motivated by how manual gating is used to identify specific cell types in flow and mass cytometry; cell types are identified through series of binarization of marker expression, such as CD3 positive and CD4 positive resulting in identification of CD4 T cells. In the ‘binary’ mode, we first binarize the data using the StepMiner [22] and then the distance between two data points across the two datasets are defined by counting the number of markers that are both positive (or negative) in the two datasets.

**Fig 3:**
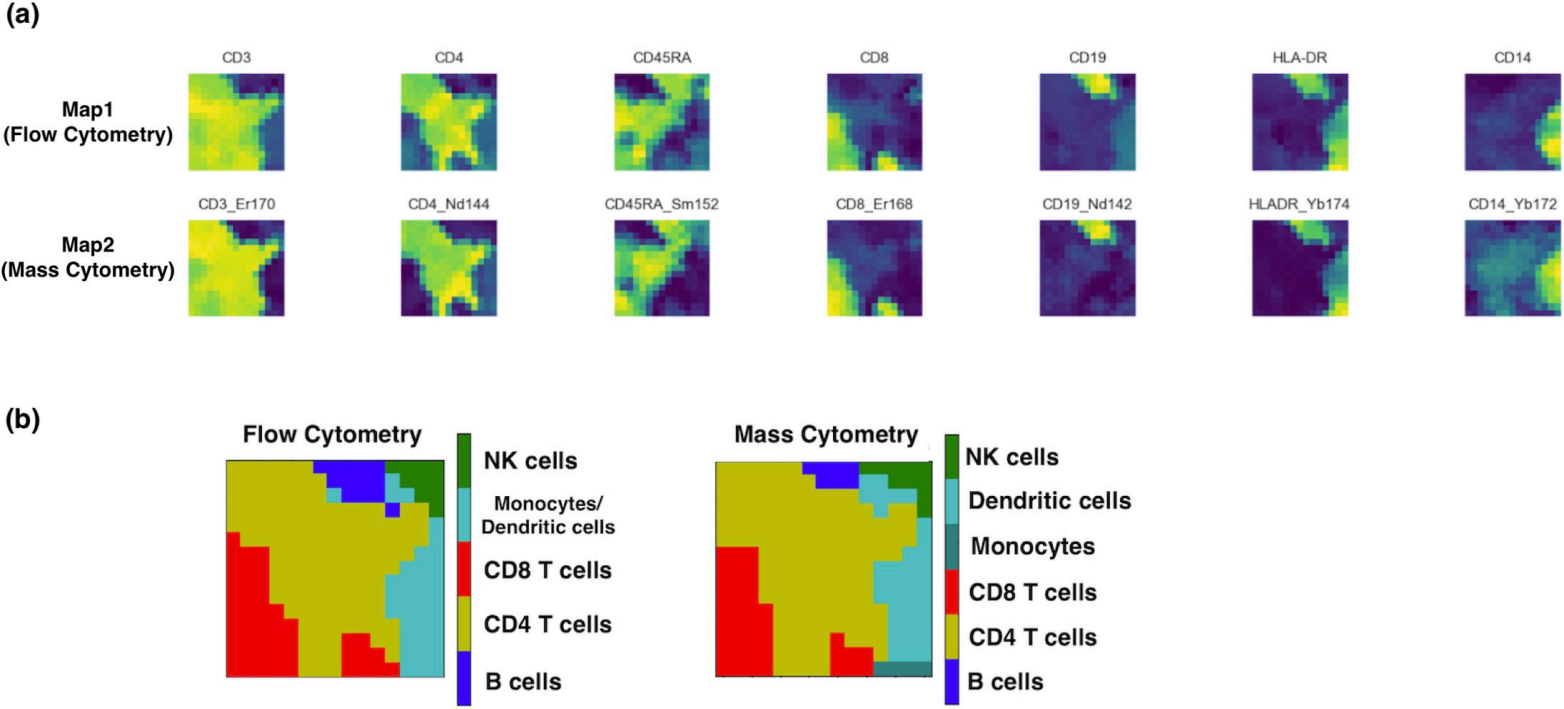
Visualizations of Features and Majority Cell Types of JSOM Maps. (A) Visualizations of the 7 features shared by the two datasets. The top row visualized the JSOM map based on the flow cytometry dataset, and the bottom row was based on the mass cytometry dataset. JSOM successfully aligned the two dataset, as manifested by similar color patterns in the two rows. (B) After data points were assigned to nodes, every node’s majority cell type was visualized through the ‘mode maps’. Different colors represented distinct cell types. Two mode maps shared similar color landscapes suggesting the two datasets were correctly matched via JSOM.

The two datasets were used to align 15-by-15 JSOM maps. Upon training with JSOM, all data points/cells in the two datasets were assigned to nodes in their respective maps by finding the nearest nodes. To confirm how well the maps are aligned, we provided visualizations of the 7 shared features of the two datasets in Fig 3A. Maps on the first row are visualizations of the features for the map corresponding to the flow cytometry data, and the bottom row corresponds to the mass cytometry data. This figure clearly shows that the shared features were aligned across the maps. It is worth noting that here, although we only showed the 7 features that were shared between the two datasets, each map actually contained each dataset’s original number of features, which was 35 and 48 respectively. Such preservation of the original features promoted robust clustering while the shared features promoted robust matching between the two datasets. After data points from each dataset were assigned to their closest nodes, we identified the majority cell type assigned to each node, and displayed the results using what we call the ‘mode maps’ in Fig 3B. Here different color represented distinct cell types. The same colors occupied similar regions in the two mode maps, suggesting that the maps were well aligned, and that at each node level, the cells belonging to the same-location node are indeed of the same/similar cell types. As quantitative measures of JSOM’s performance in aligning these two datasets, the matching score for this analysis was 0.882, and the clustering score is 0.988.

**Fig 4:**
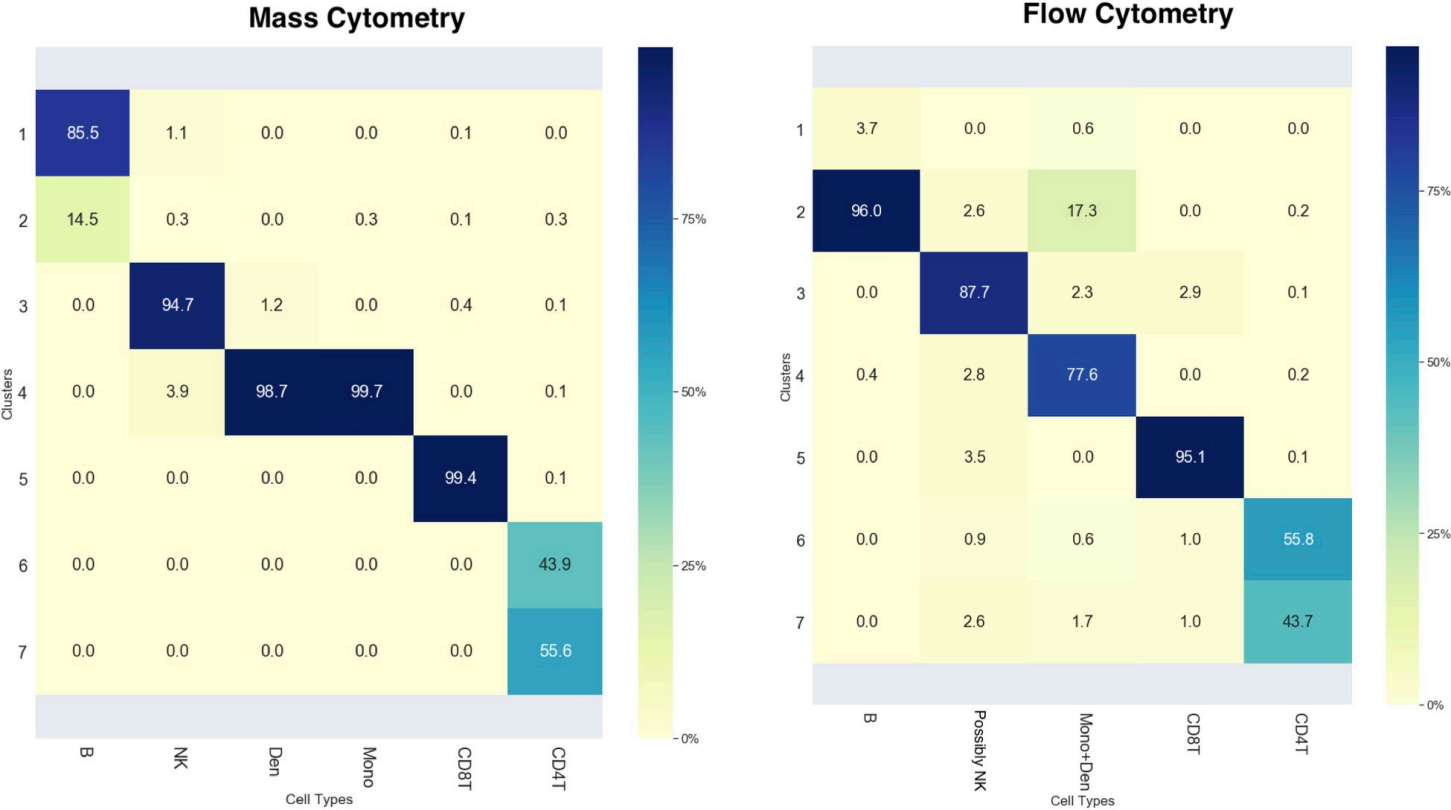
Superposition and Hierarchical Clustering of JSOM Aligned Flow and Mass Cytometry Datasets. The tables summarized the compositions of the 7 cell clusters derived by hierarchical clustering of superposition (concatenation) of the two sets of vectors of the JSOM nodes. Each column showed the distribution of one manually gated cell type across the 7 clusters.

As briefly introduced in the overview of JSOM section, JSOM could provide another insight for biological interpretation of the cellular heterogeneity in the data through superposition of two maps—concatenating two vectors of the same location node—and performing the hierarchical clustering of nodes using the concatenated vectors. Users could choose the granularity of the clustering by defining number of clusters they want to see. Applying this, we further clustered the nodes into 7 clusters. Superimposed nodes were clustered by hierarchical clustering with Euclidian distance and Ward’s linkage. We then provided a list of clusters and data assigned for nodes belonging to each cluster from the both datasets. Fig 4 summarizes the proportions of various cell types for each of the 7 clusters. For both datasets, clusters 1~2 covered vast majority of B cells, and cluster 3 were assigned with majority of ‘possibly NK cells’ from flow cytometry and NK cells from mass cytometry. Clusters 4 was assigned with monocytes and dendritic cells from both datasets, and cluster 5 ~7 covered the CD4 and CD8 T cells. It is worth noting that each cluster was primarily dominated by one cell type reflected by each row having only one element that had a significant percentage, showing that JSOM effectively separated the cell types while matching/aligning the datasets. Furthermore, it is important to point out that the ‘possibly NK cells’ group in the flow cytometry dataset was matched with NK cells from the mass cytometry dataset, suggesting that the conjecture we made about NK cells in our gating analysis of the flow cytometry dataset was true.

### scRNA-seq datasets from different technologies

Lastly, we applied JSOM to two scRNA-seq datasets acquired by two distinct technologies. As mentioned previously, recent advances in scRNA-seq have generated multiple experimental technologies and protocols that are used by biologists to investigate cellular heterogeneity of gene expression, and JSOM could facilitate comparisons of datasets generated by different technologies and protocols. Here, the two datasets examined by JSOM were one Peripheral Blood Mononuclear Cells (PBMC) dataset generated by 10X Genomics sequenced on the Illumina NextSeq500, and another PBMC dataset acquired by a technology called Seq-Well introduced by Gierhan et al [4].

The 10X Genomics dataset contained scRNA-seq counts for 32,738 genes in 2,700 single cells, and we identified 6 distinct cell types using Seurat, which is a well-established scRNA-seq analysis tool [3]. The Seq-Well data contained gene expression counts for 6,713 genes in 4296 single cells with 7 distinct cell types, of which 6 were overlapped with the 10X Genomics dataset. The cell types were identified using the same computational analysis pipelines used in the original paper [4]. To handle the high-dimensionality of the scRNA-seq data, we used the standard preprocessing workflow similar to Seurat. For each dataset, we excluded cells that have unique gene counts over 2,500 or less than 200, and cells that have greater than 5% mitochondrial gene counts. Separately for each dataset, we identified highly variable genes using the variance-to-mean metric, followed by dimension reduction using PCA to keep the top significant principle components defined by permutation analysis [3]. After the preprocessing pipeline, the 10X Genomics dataset reduced to 2638 cells with 20 PCA features, and the Seq-Well data reduced to 4214 cells with 30 PCA features. Since the PCA-based dimension reduction was performed separately to the two datasets where the lists of highly variable genes were different, the 20 and 30 PCA features in the preprocessed datasets were not directly comparable.

To generate the datapoint-to-datapoint matching functions needed for JSOM, we decided to use simple correlation based on the overlap between the lists of highly variable genes in the two datasets. There were a total of 301 common highly variable genes between the two datasets. We used the Pearson’s correlation to construct the datapoint-to-datapoint matching function, which matched each cell in one dataset to the most correlated cell in the other dataset, and vice versa, similar to the matching function in the previous subsection on flow cytometry analysis.

**Fig 5:**
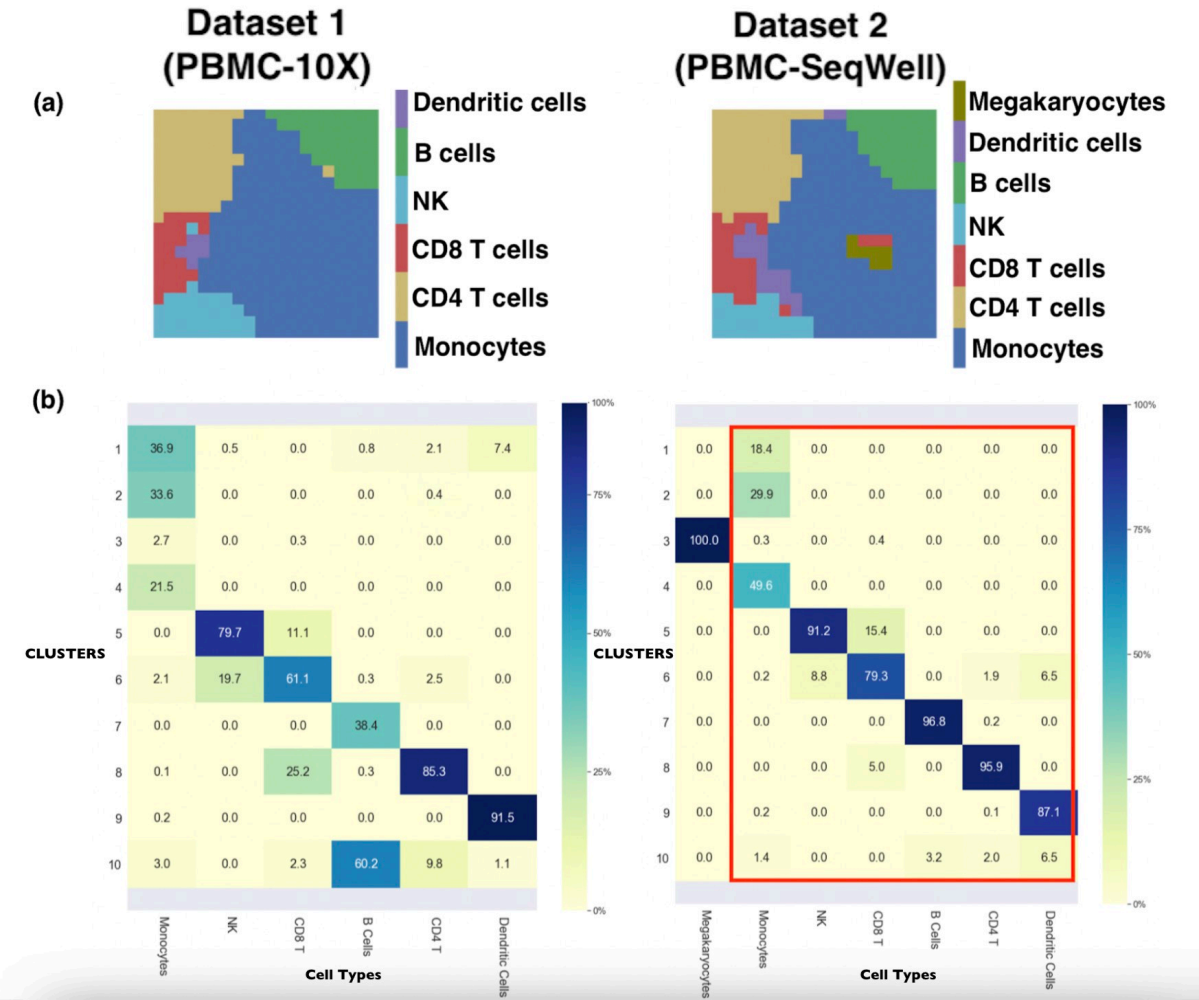
JSOM Results on Aligning Two scRNA-seq Datasets. (A) After JSOM training and assigning data points to nodes, we visualized the JSOM mode maps for the two datasets, showing the regions each cell type occupied in the JSOM map. The similar color landscape of the two visualizations suggested that JSOM successfully aligned the two datasets. (B) The tables summarized the composition of the 10 cell clusters derived by hierarchical clustering of the JSOM superposition of the two datasets. Each column showed the distribution of cells in one cell type across the 10 clusters. The red box indicated cell types shared by both datasets. Megakaryocytes which was only present in the second dataset exclusively occupied one cluster, and only a small proportion of monocytes from the first dataset is assigned to the same cluster. The datasets were correctly aligned as seen by the same cell types assigned to the same clusters.

With the preprocessed datasets and the datapoint-to-datapoint matching function generated by the common highly variable genes, JSOM was applied to align the datasets and identify common cell types. JSOM maps were trained with 20 and 30 PCA features, because the smaller number of features could prevent problems caused by the curse of dimensionality. Fig 5 summarizes the results of the JSOM analysis. Upon training with JSOM, all data points/cells in the two datasets were assigned to nodes in their respective maps. We then provided visualizations of the mode maps (Fig 5A). It can be observed that the same cell types in the two datasets occupied the similar regions of their respective maps, which are manifested by similar color pattern across the maps. The matching score for this analysis was 0.887, and the clustering score is 0.964. Once again, the two maps were superimposed, and the hierarchal clustering was applied to the concatenated vectors for clustering the nodes. Here, the hierarchal clustering led to 10 clusters, which were summarized in Fig 5B. Monocytes from both datasets were clustered into the first 4 clusters, and NK, CD4 T, CD8 T, B and dendritic cells were almost exclusively assigned to one or two clusters. Notice that the Seq-Well dataset contains megakaryocytes that were not present in the 10X Genomics dataset. All megakaryocytes were captured by a single cluster, cluster 3. Also, both monocytes and megakaryocytes are derived from the common myeloid progenitor, and the mode map shows that megakaryocytes are located within monocytes. Despite the difficulties stemming from different technologies used for the generation of datasets, JSOM showed it could robustly align maps and identify related clusters.

### Application as label transfer

Label transfer refers to the projection of annotated cell labels of a reference dataset to a query dataset. Label transfer is especially useful in single-cell data analysis where labeling of cells require non-trivial amount of efforts and background knowledge. Label transfer comes in conjunction with data integration because transferring of label is infeasible if datasets are not integrated or aligned correctly. Recent developments of the Seurat package included data integration pipelines for integrating various modalities of single-cell sequencing data using ‘anchors’ that identify pairwise correspondence between cells across datasets [23], and can be applied to perform label transfer.

The aligned maps of JSOM could also be used for label transfer. Once the JSOM maps are trained to align two datasets, and data points from the two datasets are assigned to nodes, we have information of how cells are assigned from the first dataset, and how are from the second dataset. If we assume cells from the first dataset as the reference dataset for which we know their labels, the mode defined by the reference dataset could be ‘transferred’ to label cells from the second dataset that we do not know the cell labels. In fact, this is how matching score is evaluated throughout the series of analyses above. To quantify JSOM’s label transfer capacity, we compared our results from aligning the two scRNA-seq datasets from different technologies by applying the same datasets to label-transfer function from SeuratV3 [23]. The label transfer accuracy of Seurat was 82.03%, whereas the accuracy of JSOM’s 88.7%. The comparison indicates that JSOM’s label transfer capacity is comparable with the current state-of-the-art tool.

### Comparisons with other single-cell batch-effect correction methods

Combining multiple single-cell RNA sequencing datasets is becoming more prevalent in single-cell data analysis pipelines, and many methods have been developed to facilitate batch-effect removal and data integration [24]. Although the main purpose of JSOM is not for correcting batch-effect between different datasets, JSOM produces maps that are able to align related cell clusters in different datasets, and such alignment can be considered as batch-effect correction to some extent. Therefore, we compared JSOM with popular batch-effect correction methods: Seurat3, MNN Correct, and MMD ResNet [12,23,25]. These three methods were selected from a previous benchmarking study for batch-effect correction, where they showed varying levels of performance in two different datasets [24]. The first dataset consists of two batches of human blood dendritic cell scRNA-seq data [26]. The second dataset consist of data from two independent mouse cell atlas studies. Similar to the previous benchmarking study, 7 major cell types—bladder, kidney, liver, lung, pancreas, spleen, and thymus—from the mouse cell data were chosen in our comparison [27,28]. We applied JSOM and the three batch-effect correction methods, Seurat3, MNN Correct, and MMD ResNet, to these two datasets. The resulting batch corrected data were pooled together and further clustered using SOM with the same parameters used in JSOM, and the matching score was computed based on the SOM clusters generated from batch corrected data. Therefore, the batch-effect correction methods and JSOM can be directly compared using the matching score which evaluates their ability to align related cell clusters in different datasets. Higher matching score indicated better alignment of related clusters, and thus, better performance in terms of batch-effect correction. We also examined another metric inspired by kBET [29], which was used in the previous benchmarking study and aimed to evaluate batch correction performance by measuring the divergence between the local batch label distributions against the global batch label distribution. For each node of JSOM or each SOM cluster from the batch corrected data, we computed the distribution of batch labels for the cells belonging to the node or cluster, which formed a local batch label distribution, local to an individual JSOM node or an individual SOM cluster. For each method compared, we computed the Kullback–Leibler (KL) divergence between the local batch label distributions and the global batch label distribution, and the average KL divergence was used as a metric to evaluate performance. The average KL divergence score to quantifies how well batches are mixed. Lower average KL divergence indicated better mixing of the batches, and thus, better performance.

When applied to the two batches of human blood dendritic cell scRNA-seq data, JSOM’s matching score was 0.89, compared to 0.88, 0.83, and 0.04 for Seurat3, MNN Correct and MMD ResNet respectively. JSOM’s average KL divergence score was 0.9, compared to 1.47, 1.87, and 4.5 for Seurat3, MNN Correct and MMD ResNet respectively. When applied to align the two mouse cell datasets, JSOM’s matching score was 0.72, compared to 0.76, 0.79, and 0.03 for Seurat3, MNN Correct and MMD ResNet respectively. JSOM’s average KL divergence score was 0.77, compared to 0.77, 0.63, and 3.71 for Seurat3, MNN Correct and MMD ResNet respectively. The comparison among the three batch-effect correction algorithms was consistent with the previous benchmarking study [24]. The results from the comparison suggested that JSOM’s performance is comparable to the existing top-performing batch-effect correction methods developed for scRNA-seq data.

## Discussion

We propose a new algorithm, JSOM, to align two datasets through jointly evolved self-organizing maps. Once the JSOM maps are constructed, the two maps could be used to identify related clusters between the two datasets. The proposed algorithm, which is based on the popular SOM algorithm, induces the joint evolution of the low-dimensional discretized maps, and such a joint-evolving scheme enables the map to not only capture the topology of individual datasets but also align two datasets. As long as there exist features that are shared or related between the two datasets, JSOM could be used to match the two datasets, even if they are generated by different technologies. As shown in the example of aligning flow cytometry and mass cytometry datasets, JSOM could provide robust matching when there were only 7 shared features between the two datasets. Furthermore, the datapoint-to-datapoint matching function could be tailored to reflect the user’s knowledge on the relationship of the two datasets. Note that the features used to define the datapoint-to-datapoint matching function and the features used to update the JSOM vectors do not have to be the same. In the example of aligning two scRNA-seq datasets, the datapoint-to-datapoint matching function was defined by correlation based on 301 overlapping highly variable genes in the two datasets, whereas the JSOM vectors lived in a 20-dimensional PCA space and a 30-dimensional PCA space derived from the two datasets separately. This flexibility decouples (1) the datapoint-to-datapoint matching function which is intended to reflect similarity across the two datasets, and (2) the cluster and similarity structure within each dataset, which are two important aspects for data integration, but are not always captured by the same set of features in the two datasets. We also demonstrated the application of JSOM as label-transfer tool. Although we performed comparison tests for JSOM with other batch-effect correcting methods for the scRNA-seq, we believe JSOM’s strength is its versatility. We demonstrated alignment between flow cytometry and mass cytometry datasets and between two different scRNA-seq technologies, and the combinations could be extended to many different scenarios such as between flow cytometry to scRNA-seq or between scRNA-seq to scATAC-seq. There exist limitations of JSOM to be mentioned. The matching power of JSOM is to some extent subjected to the robustness of datapoint-to-datapoint matching functions that are evaluated from the shared features. JSOM will fail to provide correct matching if the quality of the initial matching function is poor or spurious. For all experiments above, the shared features along with the right modes (‘correlation’ or ‘binary’) resulted in good-quality matching functions. Therefore, special attention is needed to derive the matching functions—through choice of modes and choice of what related features to use—which would benefit from a user’s prior knowledge regarding the relation of the two datasets. It should also be noted that both datasets should be comprised of similar number of distinct types (labels). The number of existing cell-types was the same for the flow cytometry example, and it only differed by one for the flow-mass cytometry example and the scRNA-seq example. JSOM does put more weights on data points that have strong matching in the other dataset yet having non-overlapping types that has spuriously strong matching in the other dataset could potentially generate wrong results. Currently, the number of input datasets to JSOM was limited to two datasets at a time, yet this could be extended to three or more with modification of the algorithm and matching functions. Future studies extending from this paper would focus on improving on the limitations mentioned above for better generalizability.

## Methods

### Data preprocessing

Although JSOM could be used to align any pair of datasets, we recommend users go through proper data preprocessing that is tailored to the datasets. First, when the datasets contain a wide range of numerical values, a log-like transformation should be used to make all values in a similar scale. Such transformation is commonly recommended as a preprocessing step for many computational algorithms designed for single-cell data generated by flow cytometry, mass cytometry, scRNA-seq, etc. For all flow cytometry datasets examined in this paper, inverse-hyperbolic-sine (arcsinh) transformation with cofactor 150 was applied; for all mass cytometry datasets, inverse-hyperbolic-sine transformation with cofactor 5 was applied; for the scRNA-seq datasets, log transformation was applied. Second, if the number of features is large, the number of features in each dataset should be separately reduced through dimensionality reduction–PCA, or tSNE, etc. Such reduction enables a proper training of JSOM while preventing spurious results caused by the curse of dimensionality. Although giving a perfect threshold of the maximum number of allowable features in JSOM is impossible, the typical dimensionality in scRNA-seq datasets (i.e., around ~30,000) is too large for JSOM to handle. In the example of aligning two scRNA-seq datasets, we reduced the two datasets to 20 and 30 PCA dimensions respectively.

### Overview of JSOM

Procedures of running JSOM could be divided into three different parts: (i) Initialization part where datasets and their datapoint-to-datapoint matching are evaluated, and parameters used for JSOM are determined, (ii) training of JSOM and (iii) post-training part where data are assigned to nodes, and concatenated nodes are further clustered.

(i) Initialization. JSOM requires the following inputs from the user: two datasets to be compared (dataset1, dataset2), and two derivative datasets that contain the same/related features in the same order as seen from Fig 1. The matching function will be evaluated using the derivative datasets. Along with the matching function, each data point’s matching weight, *δ*, is measured. For correlation method, *δ* is the actual value of the correlation between a datapoint in one dataset and its matched datapoint in the other dataset. For the binary method, *δ* is the number of the co-occurring (both positive or both negative) features out of total number of features. Additionally, users could choose the number of nodes for each axis of the map, *m* and *n* respectively, and the total number of epochs for the training. A matching function contains a series of indices of each datapoint’s most related datapoint from the other dataset. Two grids of *m* by *n* nodes are generated, and each node is assigned with a vector: a vector with a length corresponding to the number of features of either dataset1 or dataset2. The values of all vectors are randomly initialized at the beginning. There are two additional parameters that are optional: *α*, and *ε*. The *α* parameter is a learning rate, which is initially set to 0.9 as a default, and it steadily (linearly) decreases as iteration continues. The *ε* parameter, which determines a radius used to determine the neighboring nodes to be updated, and also reduces as iteration continues until only one node is updated. Please refer to the original SOM for more details about these parameters. The suggested number of epochs is three, which is the default setting of JSOM, and this allows each datapoint to be used at least three times in the training process of the JSOM maps.

(ii) Training of JSOM. If size of dataset1 or dataset2 is greater than 10,000, JSOM randomly samples 10,000 data points from each dataset. The down-sampleing is performed using the density-dependent down-sampling algorithm introduced in Qiu et al. [21], and the down-sampling method aim to equalize the density of data points among various populations in the data, so that equal representation of rare and abundant populations is achieved. Users could choose to skip the density-dependent downsampling. The training of JSOM alternates between independent updating and joint updating. Below are detailed descriptions of the operation of JSOM.

For the independent updating, a datapoint ($X_{i}^{\left(1\right)}$) from the first dataset (*X*^(1)^) is chosen. For the chosen datapoint, its Euclidean distance to all the nodes of the first map ($V_{j}^{\left(1\right)}$), where *j* = 1⋯m*n, is measured. Among the nodes, the nearest node with the smallest distance from the datapoint is found. Based on the nearest node, find all neighboring nodes that are within *σ* distance from the nearest node. For iteration *t*, the neighboring nodes that meet the condition along with the nearest node are updated according to the following formula: ${V_{j}}_{t+1}^{\left(1\right)}={V_{j}}_{t}^{\left(1\right)}+\delta_{i}*\alpha *(X_{i}^{\left(1\right)}-{V_{j}}_{t}^{\left(1\right)})$. Similarly, the same procedures are done for a datapoint ($X_{i}^{\left(2\right)}$) randomly chosen from the second dataset (*X*^(2)^), and the nodes of the second map ($V_{j}^{\left(2\right)}$) are updated according to the following formula: ${V_{j}}_{t+1}^{\left(2\right)}={V_{j}}_{t}^{\left(2\right)}+\delta_{i}*\alpha *(X_{i}^{\left(2\right)}-{V_{j}}_{t}^{\left(2\right)})$.

To perform the joint updating, for the datapoint ($X_{i}^{\left(1\right)}$), find its matched datapoint (${\tilde{X}}_{i}^{\left(2\right)}$) from the second dataset (*X*^(2)^) according to the evaluated *matching_function1*. For the nodes that were updated via $X_{i}^{\left(1\right)}$, their same corresponding nodes in the second map ($V_{j}^{\left(2\right)}$) are updated according to the following formula: ${V_{j}}_{t+1}^{\left(2\right)}={V_{j}}_{t}^{\left(2\right)}+\delta_{i}*\alpha *({\tilde{X}}_{i}^{\left(2\right)}-{V_{j}}_{t}^{\left(2\right)})$. Similarly, For the datapoint ($X_{i}^{\left(2\right)}$), find its matched datapoint (${\tilde{X}}_{i}^{\left(1\right)}$) from the first dataset (*X*^(1)^) according to the evaluated *matching_function2*. For the nodes that were updated via $X_{i}^{\left(2\right)}$, their same corresponding nodes in the first map ($V_{j}^{\left(1\right)}$) are updated according to the following formula: ${V_{j}}_{t+1}^{\left(1\right)}={V_{j}}_{t}^{\left(1\right)}+\delta_{i}*\alpha *({\tilde{X}}_{i}^{\left(1\right)}-{V_{j}}_{t}^{\left(1\right)})$. *α and ε* decrease after one iteration of the update. Initial values for *α* is 0.9 and *ε* is $\sqrt{2}*\frac{\max\left(m,n\right)-1}{3}$, where *m* and *n* represent the grid size of the JSOM map.

(iii) Post-training data assignment. Upon the training of the map is finalized, JSOM finds each datapoint’s nearest neighboring node using either $V_{j}^{\left(1\right)}$ or $V_{j}^{\left(2\right)}$, and assign the datapoint to the nearest node. Optionally, JSOM could concatenate the two vectors ($V_{j}^{\left(1\right)}$ and $V_{j}^{\left(2\right)}$) for each node and perform hierarchical clustering of the nodes using Ward’s linkage based on Euclidean distance. Users could decide the number of clusters based on their knowledge of the datasets; otherwise, the number of clusters is set to be max (*m*, *n*). Finally, JSOM provides a list of nodes and clusters, as well as lists of indices for assignments of datapoints to nodes and lists of indices for assignments of nodes to clusters.

The source code of JSOM, along with examples, are available at https://github.com/hlim95/JSOM

### Node purity score

Node purity score quantifies how well JSOM clusters the data. Upon all datapoints are assigned to the nodes of the map, for subset of datapoints assigned to the same node, a mode of the labels is evaluated and the number of datapoints with labels equal to the mode is counted. Finally, node purity score quantifies the proportion of datapoints whose labels are equal to the mode of the nodes. The score ranges from 0–1, with 1 means a perfect clustering of datapoints of the same label into nodes in JSOM. The pseudocode for calculation of the node purity score is described below.

*For each node*,

*Labels1*: = *labels of data assigned from Data1*

*Labels2*: = *labels of data assigned from Data2*

*Mode1*: = *mode(Labels1)*

*Mode2*: = *mode(Labels2)*

*Purity_score* = *count(Labels1 = = Mode1) + count(Labels2 = = Mode2)*

*Purity_score* + = *Purity_score*

*Final_Purity_score* = $\frac{Purtiy\;Score}{len(Data1)+len(Data2)}$

### Matching score

Matching score quantifies how well JSOM aligns two datasets. Upon all datapoints are assigned to the nodes of the map, for the two subset of datapoints assigned to the same nodes in both maps, a mode of the labels from one dataset is evaluated, and the number of datapoints with labels equal to the mode from the other dataset is counted. Matching score quantifies the proportion of datapoints whose labels are equal to the mode of the nodes determined from the first dataset. The score ranges from 0–1, with 1 means a perfect matching result. The pseudocode for calculation of the matchings score is described below.

*For each node*,

*Labels1*: = *labels of data assigned from Data1*

*Labels2*: = *labels of data assigned from Data2*

*Mode1*: = *mode(Labels1)*

*Mode2*: = *mode(Labels2)*

*Matching_score* = *count(Labels1 = = Mode2) + count(Labels2 = = Mode1)*

*Matching_score* + = *Matching_score*

*Final_Matching_score* = $\frac{Matching\_score}{len(Data1)+len(Data2)}$
